# Public health work in Sweden during the COVID-19 pandemic: Karin Guldbrandsson

**DOI:** 10.1093/eurpub/ckac129.006

**Published:** 2022-10-25

**Authors:** K Guldbrandsson, A Månsdotter

**Affiliations:** 1 Living Conditions and Lifestyles, Public Health Agency of Sweden, Solna, Sweden; 2 Department of Epidemiology and Global Health, Umeå University, Umeå, Sweden

## Abstract

**Background and objectives:**

This study examines the consequences of the COVID-19 pandemic for public health practice carried out at local and regional levels in Sweden. The work includes, for example, interventions in health care, schools and preschools, social services, and non-profit organisations.

**Methods:**

By means of written questions and interviews in municipalities, regions, county administrative boards, networks, and organisations, we investigated whether public health-related interventions had decreased, increased, or changed as a result of the COVID-19 pandemic. Data were analysed by content analysis.

**Results:**

The results show that a large number of interventions from a variety of local and regional actors aimed at broad target groups were cancelled or paused during the time of our survey. Eventually, many, but not all of the cancelled interventions were replaced with other options, most of which are included in the following themes:

• Digital solutions and support over the phone instead of physical meetings.

• Outdoor activities instead of indoor activities.

• Organisational adaptations, for example, from drop-in visits to booked appointments and from open activities to scheduled visits.

The interviews also revealed that public health issues had been highlighted and that existing collaboration structures were a success factor in managing the consequences of the COVID-19 pandemic. The risk of and concern for the spread of infection and compliance with the authorities’ recommendations were stated to be the main reasons why public health-related interventions had decreased, increased, or changed.

**Conclusions:**

Both general public health practice and targeted interventions in health care and municipal activities have been cancelled or rescheduled according to our survey. Because many public health-related interventions have an equalising effect on health, this can be of great importance for groups that are socially, economically, or health-relatedly vulnerable.

